# The Effects of E-Cigarette Aerosol on Oral Cavity Cells and Tissues: A Narrative Review

**DOI:** 10.3390/toxics10020074

**Published:** 2022-02-06

**Authors:** Paweł Szumilas, Aleksandra Wilk, Kamila Szumilas, Beata Karakiewicz

**Affiliations:** 1 Department of Social Medicine and Public Health, Pomeranian Medical University, 71-210 Szczecin, Poland; pawel.szumilas@pum.edu.pl (P.S.); beata.karakiewicz@pum.edu.pl (B.K.); 2 Department of Histology and Embryology, Pomeranian Medical University, 70-111 Szczecin, Poland; aleksandra.wilk@pum.edu.pl; 3 Department of Physiology, Pomeranian Medical University, 70-111 Szczecin, Poland

**Keywords:** e-cigarettes, e-aerosol, chemical components, toxins, microbiome, saliva, oral cavity tissues

## Abstract

A wealth of research has comprehensively documented the harmful effects of traditional cigarette smoking and nicotine on human health. The lower rate of exposure to harmful chemicals and toxic substances offered by alternative electronic smoking devices (e-cigarettes, vaping, etc.) has made these methods of smoking popular, especially among adolescents and young adults, and they are regarded frequently as safer than regular cigarettes. During vaporization of these so-called e-liquids, toxins, carcinogens and various other chemical substances may be released and inhaled by the user. Data on the potential human health effect attendant on exposure to e-vapor are based mainly on animal and in vitro studies. The oral tissues are the first locus of direct interaction with the components of the inhaled vapor. However, the short-term as well as long-term effects of the exposure are not known. The aim of the review is to briefly present data on the effects of the chemical components and toxins of e-cigarette vapor on oral cavity cells and tissues of oral health.

## 1. Introduction

Despite there being extensive information available on the harmfulness of conventional cigarette smoking, electronic cigarettes (e-cigarettes) are regarded as a safer alternative, which are particularly popular among both adults, young adults and teens, and their use has increased rapidly [1]. Furthermore, waterpipe and cigar smoking are also alternatives to traditional smoking. A very high prevalence of smoking indicates that there is a need for smoking cessation programs, access to effective quitting treatments and mass media campaigns to diminish smoking among the youth [2]. The results from the systematic review and meta analysis performed by O’Brien group (2021) indicated that e-cigarette use in Europe and North America by teenagers correlates with the initiation of conventional smoking [1]. It was observed that vaping can be a reason for relapse into traditional smoking [3]. Moreover, it was found that third-generation electronic cigarettes may cause adverse effects in the oral cavity, and normal e-cigarette use, which involves repeated use of the same atomizer to generate aerosol, may enhance the potential toxic effects of third-generation e-cigarettes [4].

Similar results were observed among eastern European populations; additionally, a higher prevalence of e-cigarettes was recognized among males, adolescents and young adults within populations of countries in this part of the Europe [5]. In Poland, there have been studies aimed at assessing patterns of e-cigarette use and comparing nicotine dependence among cigarette and e-cigarette users in a group of highly educated young adults. The findings from two representative groups suggested that e-cigarettes may have a higher addictive potential than traditional cigarettes among young adults [6]. The next studies performed in five Polish Universities with 7324 participants showed that e-cigarette use among young adults was significantly higher in correlation to the general population [7].

Most traditional cigarette smokers who quit smoking believe e-cigarettes to be less harmful than regular cigarettes. However, the statement that e-cigarette use helps users to quit smoking is highly controversial [8].

The use of e-cigarettes (also known as “vaping”) has thus seen an unprecedented increase worldwide [9]. There are four generations of e-cigarettes, all of them are battery-operated devices and cartridge-based products containing fluid with varying levels of nicotine and flavouring and several toxicants as heavy metals [10,11,12].

While the harmful effects of traditional cigarette smoking on human health is well researched, knowledge of the effects of exposition to e-aerosol is limited, and above all there is a lack of long-term studies. The first contact of the various chemicals inhaled with the e-aerosol takes place in the oral cavity, and conclusions on the effects of this interaction come mainly from animal or in vitro studies. Additionally, conflicting results have been reported, corresponding to the various device and e-liquid combinations and different methods of study [13]. Moreover, together with the increase in EDNS popularity, many accidents were reported connected with burns of patients with various degrees as a result of e-cigarette explosions, including injury of the oral cavity [14,15]. The aim of this review is thus to present the current knowledge of the influence of the toxic and chemical components of e-cigarette aerosols on periodontal tissues and oral health. We aimed to examine articles and reports that we have evaluated, compared and described. MEDLINE, EMBASE, PUBMED, internet websites of research articles and reference lists were searched to identify articles for inclusion. Descriptive analysis was conducted.

## 2. Chemical Components and Toxins of E-Cigarette Aerosol

Vapes, vaping, vaporizers, vape pens, electronic cigarettes, e-cigarettes and e-cigs are synonyms used to describe electronic nicotine delivery systems (ENDS), which are non-combustible tobacco products. In these products, a solution of “e-liquid” held in a cartridge is heated to a temperature of 100–300 and over to 350 °C depending on the ENDS category, e-liquid composition and power output [16,17] to create an aerosol that is inhaled by the users [18,19]. Standard electronic cigarette liquid is typically composed without/with nicotine (at different doses, from 0 to 24 mg/mL), water, propylene glycol (PG), vegetable glycerin (VG) or glycerol and flavouring constituents [20]. In general, it contains at least three major ingredients: psychoactive agents as nicotine, solvents and flavouring compounds. Nicotine in aqueous solution can be found in three forms as diprotonated, monoprotonated and unprotonated. The content of diprotenated nicotine is low and its presence does not matter much in the e-fluid [21]. Therefore, two of the three forms are taken under consideration. The unprotonated—nicotine freebase is easily vaporized form, and the protonated form—nicotine salt is present in e-liquid as a product of combination of nicotine and different acids, including glycolic, pyruvic, lactic, levulinic, fumaric, succinic, benzoic, salicylic, malic, tartaric and citric acids [22,23]. The unprotonated, freebase nicotine in e-liquid is used in traditional e-cigarettes, while the newer e-cigarette generation called “pod-mods”, such as JUUL and others, use the protonated formulation derived from nicotine salts. In the e-fluid of the “pod-mods”, nicotine can be found in concentrations 2 to 10 times higher than in traditional e-cigarettes, and in high concentration of 65.2 mg/L [21,22,24]. 

The main two constituents are present in commercial refill liquid and e-liquid, propylene glycol odorless and tasteless, and vegetable glycerin with a warm, sweet taste that are used as a solvent in e-liquid. The ratio of PG and VG content is perceived by the producers and users of e-cigarettes as an important determinant of their sensory characteristics [25]. Together with the propylene glycol, glycerol and nicotine, the e-liquid also contains various flavors [18]. The flavoring compounds have different names and aromas, such as strawberry, root beer, chai tea, chocolate, fresh watermelon, black currant, forest berries, cherry, and grape; this variety of flavors is one of the main factors that increases attraction for new users, especially for adolescents [26].

During e-liquid heating, aerosol is formed and the main components PG and VG can be disintegrated into hazardous toxic substances. The content and amount of which depend on the type of the device, brands and the smoking parameters. There are toxicants, such as carbonyls, formaldehyde, acetaldehyde, acrolein, crotonaldehyde, epoxides and glycidol in aerosol after the thermal decomposition of PG and VG. The products that are produced during the heating of fluid were divided into three groups: (i) thermal decomposition products derived from PG and VG; (ii) products that originated from other compounds; and (iii) products formed directly [18].

The study of Khlystov and Samburova (2016) with various e-cigarette brands indicated that during the heating of the flavouring compounds, the formation of aldehydes dominated, and these included formaldehyde, acetaldehyde and acrolein [26]. Other research has supported these results, with an emphasis on their potentially harmful effects for human health [27,28]. Nevertheless, multiple potentially harmful components, toxic metals and trace elements, e.g., aluminium, lead, mercury, zinc, carbonyls, epoxides, policyclic aromatic hydroxycarbons (PAHs) and pesticides were found in e-liquids and aerosols [29,30,31,32,33]. New methods used to estimate toxic metal-containing particles in e-aerosols of various pod-type systems are permitted to detect metal-containing particles such as chromium, zinc, iron, cooper, tin, and lead in various concentrations [34]. Recently, Tehrani et al. (2021) performed nontarget and quantitative analyses of e-liquids containing nicotine and generated e-aerosols from four of e-cigarettes: one disposable, two pod and one tank/mod to identify earlier unknown compounds. The liquid chromatography-high-resolution mass spectrometry (LC–HRMS) and chemical fingerprinting techniques used in the study permitted to observe that the number of detected compounds increased significantly in e-aerosol compared to e-liquid in three tested types of e-cigarettes [35].

It has also been showed that aerosols produced during the heating of e-liquids contain reactive oxygen species (ROS) and can produce oxidative stress through the presence of free radicals, NO [36,37] and carbonyls [38]. It can be suggested that falvoring chemicals and nicotine play an important role in the production of ROS [39,40,41], and the amount and proportions vary from product to product [42]. Nevertheless, the studies also showed that the levels of free radicals are lower in e-fluid and the gas phase of aerosol and heat-not-burn products compared to traditional cigarettes, and contain fewer toxic substances at lower concentrations [43]. An exposure of tissues to free radicals can result in damage to the proliferation, survival and inflammation pathways in cells [36].

It is well-documented that cigarette smoking is considered as a risk factor for inflammatory airway diseases and chronic obstructive disease [44,45]. The components of cigarette smoke can affect transcriptome alteration through chromatin remodelling and DNA methylation in the cells of the respiratory system. These changes are essential on the level of DNA and of specific genes [46]. The use of e-cigarettes can also be expected to have similar harmful effects. These inhaled substances are also classified as toxic and hazardous, particularly for the respiratory system, where they can disturb the oxidative–antioxidative balance (free radicals, irritants) [11,20,47]. e-cigarette aerosols may be expected to cause genotoxicity and immunotoxicity, but to lesser degrees than cigarette smoke. Some of the chemical substances detected in the fluid/aerosol of e-cigarettes or in heat-non-burn products are listed in Agents Classified by the IARC Monographs (Table 1) as cancerogenic to humans (group 1), probably carcinogenic to humans (group 2A) and possibly carcinogenic to humans (group 2B) [48].

Therefore, the need to use modern analytical, reliable and validated methods to quantify both toxic metals and other chemicals inhaled by users of various generations of e-cigarettes, especially the long-term effects of exposition to the compounds, are not fully known. A variety of components seem to indicate that vaping should not be treated as a safe alternative method compared to traditional smoking.

## 3. Effect of E-Cigarettes Aerosol on Oral Cavity

It is known that both environmental and civilizational factors can affect human health and can impact the functions of tissues and organs. One factor is cigarette smoking and its harmful effects on human health are well documented. Although the content of various chemical compounds and trace elements in e-aerosol are described to be lower than in cigarette smoke, long-term exposure to aerosol can have a negative effect on oral cavity health [16]. However, there is an increasing amount of data on the risks of e-cigarette use compared to the benefits. Vaping, an alternative that simulates tobacco smoking, involves the inhalation of aerosols created by heating of e-cigarette fluid, often considered less harmful [49,50]. The National Academy of Sciences, Engineering and Medicine published in 2018 a report reviewing the evidence for the adverse effects of e-cigarettes in the course of oral cavity and respiratory system diseases [49].

The tissues of the oral cavity are those first exposed to the inhaled e-aerosol and they interact directly with its toxins and chemical components. Research on potential oral health changes following e-cigarette exposure is limited and there is some controversy about the safety of e-cigarette use [51], and daily vaping is associated with poor oral health [25].

Experimental studies are not able to fully reflect real conditions because regular electronic cigarette users may draw more puffs a day than in experiment laboratory studies [52,53]. To evaluate the effects of e-aerosol exposure on the human oral cavity, the development and severity of periodontal disease should be taken into consideration, such as bleeding from gingival tissue after probing, the assessment of the amount of plaque (plaque index), the quantification of the gingival crevice as a marker of periodontitis and the potential effects on the lining of epithelial cells and the oral microbiome [49,50].

### 3.1. Oral Microenvironment

The maintenance of homeostasis and functionality of the oral cavity are created by saliva, the fluid secretory product of major and minor salivary glands. There are three major paired salivary glands located outside the mouth and their secretion is transported via ducts opening in the oral cavity. The minor salivary glands are located in the mucosa and submucosa of the oral wall. Saliva with a unique composition plays protective and digestive functions, containing water, electrolytes and various protein and signaling molecules [54].

One pilot cross-sectional study was performed with volunteers to assess the effect of e-cigarette use on biomarkers of inflammation, oxidative stress, anti-inflammatory lipid mediators, tissue injury and repair and growth factors in saliva and gingival crevicular fluid. The obtained results were compared between four groups of participants as e-cigarette users (EC), non-smokers, cigarette smokers and both e-cigarette and cigarette (dual) smokers. There was significant increase between levels of myeloperoxidase and matrix metaloproteinase-9 in EC vs. non-smokers, and between dual smokers and EC in inflammatory mediators as receptor for advanced glycation end products, myeloperoxidase and recombinant human uteroglobin/CC10 [55].

The changes in antioxidant capacity of saliva in e-cigarette users and cigarette smokers comparing to non-smokers were observed in a study by Cichońska et al. (2021) [56]. The uric acid, hypoxanthine, xanthine, TAOS (total antioxidant status) and TEAC (Trolox equivalent antioxidant capacity) were determined in the samples of saliva patients. The antioxidant capacity of saliva was affected in the e-cig users in a similar degree as in cigarette smokers when compared to the saliva of non-smokers [56]. The impaired antioxidant function of saliva can stimulate the formation of free radicals and reactive oxygen species (ROS), which play a role in the progression of periodontitis and destruction of tissue [57].

### 3.2. Oral Microbiome

The microorganisms, harboring over 700 species, that reside in the human oral cavity are described as the oral microbiome, oral microflora or oral microbiota [58]. The microorganisms play an important role in the maintenance of the proper environment in the oral cavity and encompasses oral niches, such as teeth surface, tongue, cheeks, subgingival and supragingival plaque, palates, tonsils and salivary [59,60]. A dysbiosis, an imbalance in the microbial ecosystem, can produce changes in their functional composition and can result in pathological conditions [59,61,62,63]. The flavoring compounds of e-liquids have various aromas and tastes, and some chemical components, such as saccharides and sucralose are added to provide a sweet taste [64], they may selectively disrupt the homeostasis of the oral microbiome and can be associated with a variety of oral diseases, such as periodontitis and caries. There is evidence that e-cigarette use is associated with a compositional and functional shift in the oral microbiome, with an increase in opportunistic pathogens and virulence traits [65].

The study of salivary microbial communes performed by Pushalkar et al. (2020) revealed the alterations in the content and differences in oral microbiota of e-cigarette users compared to those who have never smoked. They included significantly altered beta-diversity in species and the most abundant species of bacteria *Porphyromonas* and *Veillonella* in e-cigarette users. Additionally, levels of cytokines (IL-6 i IL-1β) were increased. The dysbiosis in microbiome was related to elevated proinflammatory cytokines release and increased inflammation, which clearly indicated that e-cigarette users are more prone to infection processes in the oral cavity [66]. Alterations in bacterial taxonomic composition were also found in buccal samples and the samples of saliva in e-cigarette users. The saliva of the users presented a significantly higher alpha diversity, which declined together with decreased use of e-cigarettes. The most abundant bacteria genera in buccal samples was *Streptococcus* in both e-cigarette users and non-smoking/non-vaping, whereas *Prevotella* was the most abundant in the saliva samples also for both tested groups. When cohorts in aggregate were tested, the buccal samples of e-cigarette users were rich in *Veillonella* and *Haemophilus* species [67].

The salivary malondialdehyde (MDA), total salivary mucins (SM) and buccal smear cells of the micronuclei (MN) were analyzed in patients of three groups: e-cigarette with/without nicotine content users and a non-smoking group as in Menicagli et al. (2020) studies. A significantly higher concentration of malondialdehyse, the final product of polyunsaturated fatty acids peroxidation [68], frequently recognized as a marker of oxidative stress [69], was observed in e-cigarette users compared to the control group. The highest, statistically significant amount of salivary mucin was noted in those smoking e-cigarettes with nicotine. Analysis of the presence of micronuclei in buccal smear cells is used as a biomarker of genotoxicity in smokers to predicting the effects of carcinogens [70]. In the studies, the micronuclei were detected in exfoliated buccal cells of e-cigarette users. However, within the e-cigarette users, there were volunteers with a higher MN score, but who have a higher age (≥39 years). All of the phenomenon could be associated with the free radicals formation and to damage the normal cellular metabolism [71].

There are reports that e-cigarettes can induce changes in epithelial cells on the molecular level that result in the deregulation of gene expression. Tommasi et al. (2019) performed whole transcriptome profile analysis of oral epithelial cells obtained from central and distal regions of the inside of each cheek of volunteers who were e-cigarette users, cigarette smokers and control non-smokers or non-vapers. Analysis of the global transcriptome profile showed the deregulation of number a key genes and molecular pathways in the oral epithelial cells. Functional pathway analysis of differentially expressed genes indicated that in both e-cigarette users and smokers the genes were mainly associated with cancer within disease and disorders. In both experimental groups, differentially expressed genes were predominantly associated with “cancer”. The canonical Wnt/Ca+ pathway in e-cigarette users and the non-canonical integrin signaling pathway in smokers were the most disrupted pathways [72]. The result showed that e-cigarette use leads to the deregulation expression of key genes and molecular pathways in oral epithelial cells that are directly exposed to carcinogens.

To explain the effects of e-cigarettes on oral cavity, a prospective cross-sectional study was conducted to evaluate the prevalence and characteristics of oral mucosal lesions in former smokers and e-cigarette users. There were no significant differences in prevalence of oral mucosal lesions between the two groups. However, more frequent symptoms and the prevalence of mucosal lesions, such as nicotine stomatitis, hairy tongue and angular cheilitis were identified among e-cigarettes users [73].

The lamina propria of the lips, cheeks, floor of mouth and the ventral surface of the tongue is lined by stratified squamous nonkeratinized epithelium. The mucosa of the regions in oral cavity is very thin and well vascularized, and it is an attractive route for the administration of drugs and other therapeutic agents. A pilot study was performed by Reuther et al. (2016) to assess the possible effect of e-cigarette use on blood flow in the buccal mucosa as a consequence of postoperative patients’ questions about whether to continuing smoking or to switch to vaping. The laser Doppler technique was used to measure the flow of buccal mucosal blood in 10 volunteers showed that e-cigarettes produced a temporary rise in capillary perfusion, which can suggest the better absorption of medicines. However, the results are needed to confirm that e-vapor can improve healing time after oral and maxillofacial operations [74].

The presented data suggest that vaping disrupts both oral microenvironment and oral microbiome.

## 4. Injury of Oral Cavity as Effect of E-Cigarettes Explosion

One of the complications of e-cigarette use is their malfunctions; spontaneous failure and intra-oral explosion can result in several serious oral injuries, such as oral hard and soft tissue injuries [14,75,76]. Examples of cases of two male patients’ injuries after the explosion of the e-cigarette in the mouth included intraoral burns, luxation injuries and alveolar fracture [77], and the other, a fracture, tearing out and dislocation of the front teeth, premaxillary fractures and permanent cuts to the upper lip, the mucosa of the lips, gums, tongue and hard palate [15]. The majority of reported electronic cigarette-related oral injuries have been serious and have frequently required the intervention of a plastic surgeon. The injuries included tooth fracture and tooth avulsion, jaw fracture, dento-alveolar fracture, haematoma formation, traumatic ulceration and tattooing, intra-oral burns and subsequent necrosis, palate perforation with extension into the nasal cavity and extensive soft tissue deficits [78,79,80]. Recently, a case report of 19-year-old boy was presented with maxillofacial injury after an e-cigarette battery-related explosion in his mouth, as a warning to the public. Significant hard and soft tissue injuries of the oral cavity, in particular the anterior left maxilla were observed. Additionally, there were epidermal burns to the facial area, including the lips and upper chest; the upper lip sustained minimal soft tissue damage [81].

Therefore, to promote the awareness of this phenomenon, wide discussion, especially among adolescent and young adult of e-cigarette users, should be carried out regarding the risk of spontaneous failure and explosion of e-cigarettes. Dental professionals have an important role to play in educating patients about not only the potential harm and the health consequences of e-cigarette use, but also risk of intra-oral explosion of e-cigarettes.

## 5. Conclusions

The study indicates that exposure to e-cigarette aerosol that contains various ingredients, toxicants and carcinogens can exert harmful effects and induce changes in human oral health, inducing disbiosis, inflammation, cytotoxicity and genotoxicity, contributing to in periodontal diseases. The mechanisms of action of the chemical substances in e-aerosols (vapors) include changes on the biochemical, cellular and molecular levels. However, there is a need for extensive research to assess the actual effects of e-aerosol on oral cavity tissues as well as to evaluate the short-term and long-term use of e-cigarettes and related products. These data illustrate the need for the monitoring of the conditions of different tissues and organs of the e-cigarette users. The obtained results may be important for both adolescents and young adults.

## Figures and Tables

**Table 1:** Agents that can be found in e-aerosol, e-fluid and released from metallic coils [48].

CAS Number	Component	Group
75–07-0	Acetaldehyd	2B
107–02-8	Acreolin	2A
50–00-0	Formaldehyde	1
7439–92-1	Lead	2B

CAS (Chemical Abstracts Service).

## Data Availability

Data available in a publicly accessible repository.
